# Enhancing single shot unsupervised domain adaptation for inter-camera person re-identification

**DOI:** 10.1038/s41598-026-37168-9

**Published:** 2026-04-02

**Authors:** M. K. Vidhyalakshmi, S. Neduncheliyan, A. Hemlathadhevi, S. P. Samal, S. Barick

**Affiliations:** 1 Department of Computing Technologies, SRM Institute of Science and Technology, Kattankulathur, Chengalpattu Dt, Tamilnadu India; 2 Department of AIML, Rajalakshmi Institute of Technology, Kuthambakkam, Chennai, Tamilnadu India; 3 Department of Computer Science and Engineering, Panimalar Engineering College, Poonamallee, Chennai, Tamilnadu India; 4 Department of Biosciences, Saveetha School of Engineering. Saveetha Institute of Medical and Technical Sciences, Chennai, 602105 India; 5 Manipal Institute of Technology, Manipal Academy of Higher Education, Manipal, India

**Keywords:** Single shot unsupervised domain adaptation, Cycle GAN, Median filter, Histogram equalization, Siamese network, Computational biology and bioinformatics, Engineering, Mathematics and computing

## Abstract

Inter-camera person re-identification (re-ID), is the process of identifying people in a surveillance system from various camera perspectives. It involves confirming person identification throughout several cameras and navigating limitations like transferring lighting, converting views, and occlusions all important for safety and monitoring applications. Managing differences in camera angles, occlusions, and illumination may be difficult. These elements may cause mismatches among people, which could decrease the re-ID system’s basic efficacy. This research proposed a novel technique to enhance Single Shot Unsupervised Domain Adaptation for Inter-camera Person Re-ID to address this problem, proposed work consists of Preprocessing, and Classification. Initially the preprocessing is applied via Augmentation the use of Cycle GAN, Noise reduction the use of Median Filter, and Enhance Image contrast using Histogram Equalization (HE). Using those preprocessed data, the Siamese Network is trained under the Classification stage. To further enhance the procedure inside the Siamese Network, utilize Conv50 and Conv152. The Python platform is used to develop the suggested model, and performance metrices are used to evaluate the model’s effectiveness.

## Introduction

Inter-camera person re-ID is a computer vision issue, that refers to the processes of re-identifying humans across different cameras used in surveillance systems^1^. Overcoming adjustments in point of view, lighting, image resolution, and occlusions is vital. In this regard, the purpose is to suit one particular character identified in a given surveillance community camera frame to the equal individual performing in any other camera frame with utmost accuracy^2,3^. Hence, state-of-the-art techniques like deep neural networks, metric learning, and feature extraction are usually used to solve this problem. Inter-camera person re-ID allows people to be accompanied in several locations and makes surveillance systems powerful and efficient inside the surveillance of public places and public safety^4^. It has vast programs, from protection and public protection to video analytics. Some benefits associated with inter-camera person re-ID are to enhance surveillance powers, to follow a person throughout unique places, to support law enforcement, and to increase public protection^5^. It complements real-time situational recognition and maximizes resource allocation in the absence of human monitoring. But it additionally comes with huge troubles, like lights effects, angle versions, occlusions, and scaling problems^6^.

Such technologies may give rise to issues of privacy, thus ethical and legal ramifications need to be carefully considered when weighing values of privacy versus the advancement of technology. These computer vision techniques identify or detect objects in one pass over an image or frame, not requiring several passes or iterations^7^. These techniques are appropriate for real-time applications because they are quite fast and, hence efficient in construction. One example in real-time applications is the Single Shot Multibox Detector (SSD), which uses feature maps of different sizes to predict object positions and class probabilities directly in a single step^8^. When compared to more complicated techniques, these strategies may lose some accuracy but make up for their speed and simplicity.

One goal of single-shot approaches towards person re-ID is the very quick and high-quality matching of people in an image while viewing it from several camera angles. This type of method gives high priority to simplicity and performance and generates bounding boxes and class probabilities simultaneously^9^. Though useful for real-time applications, they might give up on accuracy by resorting to much more complex techniques specifically, when managing changes in position, illumination, and occlusion. Due to the simplistic architecture, single-shot approaches toward person re-identification most often fail to successfully manage variations in illumination, position, and occlusion^10,11^. They usually depend on some fixed characteristics or attributes, which can often not capture the complexity in unique appearance^12,13^. Moreover, with other more complex approaches, their efficiency and speed may make them sacrifice in the name of accuracy^14^. Their applicability in surveillance systems with multiple cameras and gigantic data volumes may be limited due to their potential scalability issues.

To benefit from single-shot approaches toward inter-camera person re-identification, the suggested method would be using domain adaptation to improve their effectiveness. It aligns feature distributions between labeled source data from one camera view and unlabeled target data from another view by using unsupervised domain adaptation methods. This alignment helps in enhancing the accuracy of re-identification since the model generalizes better over different camera angles. Potential improvements to domain adaptation algorithms, improved feature extraction, and more regularization methods should be taken into account. Briefly, the strategy enhances the dependability and effectiveness of one-time methods for identifying individuals in multi-camera surveillance systems.

The major contributions of the paper are as follows.

- **The issue of cross-camera domain shift is specifically addressed by a novel SSUDA-based inter-camera person Re-ID framework that combines feature-level learning within a Siamese Network with data-level adaptation (CycleGAN).**
- **By combining CycleGAN-based cross-domain style transfer**,** median filtering for surveillance noise suppression**,** and histogram equalization for contrast enhancement**,** a unique**,** task-specific preprocessing pipeline is created. This configuration is designed not only for general image enhancement but also to enhance inter-camera feature consistency in Re-ID.**
- **Each branch learns complementary feature representations (mid-level and high-level) in a dual-branch Siamese architecture that combines ResNet-50 and ResNet-152. Compared to using a single backbone network**,** this paired configuration improves discriminative power for person re-identification.**
- **Selective pooling on the deeper branch (ResNet-152) is used to implement an optimized spatial feature consolidation strategy that improves the learned embeddings’ robustness and compactness under changes in pose**,** illumination**,** and occlusion.**
- **The specific integration of CycleGAN + Dual-ResNet Siamese design + enhanced preprocessing outperforms traditional single-model Siamese configurations in inter-camera Re-ID tasks**,** as shown by extensive experimental validation and ablation analysis.**

The following sections are ordered as follows: Sect. 2 explores relevant research and literature reviews, Sect. 3 introduces the proposed framework, Sect. 4 delivers a detailed analysis of the observed results and discussions, and Sect. 5 offers the final assessment of this study.

## Literature review

In 2018, Zhong et al.^15^ explained that CamStyle prevented deep network overfitting and helped smooth CamStyle discrepancies through data augmentation. Specifically, a style transfer model allowed annotated training images to be transmitted to every camera, forming an augmented training set with the original examples. This strategy increased data diversity and reduced overfitting but also introduced significant noise. To reduce noise, label smooth regularization (LSR) was used. On camera systems that frequently experienced overfitting, the vanilla approach which omitted LSR performed admirably. CamStyle has the potential to tackle challenging problems such as unsupervised domain adaptation (UDA) and one-view learning in person re-ID, which are crucial for both research and practical applications.

Multi-camera network person re-identification (re-ID) for surveillance was proposed by Liu et al.^16^, in 2020. ST-MC model predicted a person’s camera view based on statistical data on their multi-camera network entry/exit positions. The Multiple Granularity Network (MGN), the current person re-ID standard, benefited from the special expansion provided by ST-MC. Compared to CNN-only techniques, the methodology improved re-ID performance by considering contextual information as well as CNN appearance-based features. The latter complemented person re-ID with scene understanding.

Single-shot target recognition utilizing multi-scale feature fusion and feature augmentation was first shown by Qu et al.^17^,, in 2022. To improve network feature extraction, they included multi-scale feature fusion, feature augmentation, and effective channel attention modules into the detection module of the SSD target identification method. Their method worked better with many objects and occlusion, according to experimental results.

Wu et al.^18^, projected single-shot lensless imaging using Fresnel zone aperture and incoherent light in 2020. An inline hologram-like pattern was produced by the Fresnel zone aperture, which recorded incoherent rays in a wavefront-like shape. They demonstrated how the compressive sensing approach might eliminate the twin-image problem caused by ordinary backpropagation reconstruction because of the sparsity of real scenes. An extremely high signal-to-noise ratio single-shot picture reconstruction encouraged the development of a flat, dependable, and calibration-free camera design.

Feng et al.^19^, offered a collaborative learning system to learn better feature embeddings using high-precision neighbor pseudo labels and high-recall group pseudo labels to supplement these low-recall neighbor pseudo labels. To improve recall, they transitively integrated neighbors of various samples to create group pseudo labels. The merging operation might have created subgroups due to inaccurate neighbor predictions. They recommended employing a similarity-aggregating loss to draw the input sample toward the most comparable embeddings to effectively use these group pseudo labels. Their technique performed well in the unsupervised domain adaptation re-ID setting, as shown by extensive trials on three big datasets.

In 2020, Yang et al.^20^ suggested a part-aware progressive adaptation network (PPAN) for UDA-based ReID across domains using global and local relations. Under classified supply domain supervision, a multi-department network explicitly learned the illustration of discriminative features from whole-body and body-part images. An independent UDA constraint on every branch of the network aligned the worldwide and nearby feature distributions from the classified source and unlabeled target domains. Furthermore, a unique progressive adaptation approach (PAS) became used to mitigate the negative outcomes related to outlier source identity. Five datasets (DukeMTMC-reID, Market-1501, PRID, CUHK03, and VIPeR) had been used to test the proposed unsupervised ReID model, and it was found to be more reliable and effective than present strategies.

Li et al.^21^, introduced Adaptive Deep Clustering (AdaDC) to mitigate noisy pseudo-labels in 2021. The suggested method applied numerous clustering processes adaptively and instead to maximize complementary statistics and minimize overfitting noisy pseudo-labels. Integrating diverse clustering outcomes in an innovative pattern selection method reduced the noisy label ratio in pseudo-labels. Experiments confirmed that the suggested technique outperformed existing UDA person-ID algorithms on usually used datasets. Several in addition analytical experiments tested the proposed approach’s efficacy.

In 2020, Li et al.^22^, introduced a method for discriminative DIF learning by utilizing person attribute stability, complementarity, and low-level visual characteristics. The system created latent attribute-correlated visual functions and aligned person attributes to pedestrian picture nearby areas. This transformed character attributes into LAVF without area statistics to useful resource DIF learning. The technique centred on aligning human developments with local areas and enhancing function discrimination throughout pedestrian photos. To hold semantic consistency, a fully related layer changed into used for alignment between person attributes and nearby areas.

Desai et al.^23^, introduces a facial recognition approach that utilizes deep convolutional Siamese neural networks for one-shot classification and verification. It emphasizes the use of data augmentation techniques to enhance the network’s ability to generalize across varying conditions such as poses, lighting, and expressions.

Zhao et al.^24^, introduces, a novel framework that uses Vision-Language Models, specifically CLIP, to address the challenges of generalizable person re-identification. The goal is to learn fine-grained and domain-invariant features for robust person recognition across various conditions and unseen domains. Table 1 provides a comprehensive summary of the research gaps identified in the existing literature.

**Table 1:** Research gaps from the existing Works.

Author name	Aim	Method	Advantage	Research Gaps
Zhong et al^15^.	Present CamStyle as a person-ID option.	CamStyle: A style-transfer data augmentation method	• **Lessens overfitting and evens out CamStyle differences** • **CamStyle-induced noisy augmentation; restricted effectiveness on camera systems with lower overfitting**	Improves the performance of unsupervised domain adaptation and one-view learning in person re-ID
Liu et al.^16^,	Create ST-MC model for re-IDing multi-camera networks.	ST-MC predicts camera view using entry/exit point statistics.	• **Enhances re-ID performance by taking contextual information into account.** • **Restrictions on statistical data may make it difficult to manage complex scenarios or dynamic surroundings.**	Increases Rank-1 and mAP on the DukeMTMC-reID dataset significantly.
Qu et al.^19^,	Introduce multi-scale feature fusion and improvement for single-shot target detection.	SSD detection algorithm incorporates multi-scale feature fusion, augmentation, and efficient channel attention modules.	• **Increases the speed and accuracy of detection** • **Possibly difficult in situations with a lot of clutter or opacity**	With a 512 × 512 input size, detection accuracy achieves 81.9%, surpassing the original SSD technique.
Wu et al.^18^,	Suggest lensless single-shot imaging using incoherent illumination and a Fresnel zone aperture.	Fresnel zone aperture encodes incoherent photons, and compressive sensing reconstructs.	• **Eliminates the twin-image issue and encourages camera layout without calibration** • **Mostly applicable to particular lensless imaging configurations; may not work with conventional camera systems**	Reconstruction from a single-shot image with a much higher signal-to-noise ratio
Feng et al.^19^,	Provide a cooperative learning framework for unsupervised domain adaption re-identification.	Features are incorporated using high-precision neighbor and high-recall group pseudo labels.	• **Enhances feature embeddings and does well in re-ID for unsupervised domain adaption** • **The accuracy of the pseudo labels and the quality of the similarity-aggregating loss function may affect performance.**	reaches cutting-edge performance on three datasets using unsupervised domain adaptation re-ID setup.
Yang et al.^20^,	Present PPAN for cross-domain UDA-based ReID	PPAN uses global and local relations and progressive adaptation to align feature distributions across domains.	• **Greater resilience and efficacy in comparison to cutting-edge methods** • **The choice and adjustment of hyperparameters may have an impact on effectiveness.**	Using the DukeMTMC-reID, Market-1501, VIPeR, PRID, and CUHK03 datasets, performs better than current approaches
Li et al.^21^,	To lessen the detrimental effects of noisy pseudo-labels, suggest AdaDC.	Active Deep Clustering reduces noisy label ratios with adaptive clustering and progressive sample selection.	• **Attains cutting-edge UDA person re-ID performance and minimizes overfitting on noisy labels** • **Performance is influenced by the caliber and variety of clustering techniques employed**	Beats current UDA person re-ID techniques on extensively utilized datasets.
Li et al.^22^,	Identify the difficulties with unsupervised domain adaption re-ID.	Uses human and visual stability and complementarity to aid discriminative DIF learning.	• **Facilitates the acquisition of discriminative DIF and guarantees semantic coherence between local regions and attributes** • **Depending on how accurate and consistent a person’s characteristics and physical characteristics are**,** reliability might vary.**	Effectiveness proved with comparative studies on four datasets

### Problem statement

Person re-ID in surveillance systems has greatly stepped forward with current advances in computer vision. However there exist challenges with noisy pseudo-label handling, non-linear elements affecting section retrieval accuracy, and camera-related images affected by style differences. Furthermore, camera architectures that are dependable and free of calibration are still required. Scholars have suggested creative ways to close these gaps. These include single-shot BP neural network-based CFPP for high-precision phase retrieval, CamStyle for explicit management of camera style differences, ST-MC model for multi-camera network re-ID, and single-shot lensless imaging for calibration-free reconstructions. Moreover, to address issues with noisy pseudo-labels and UDA, collaborative learning systems, part-aware progressive adaptation networks, and Adaptive Deep Clustering approaches have been presented. These developments are intended to strengthen network performance, boost feature discrimination, and offer reliable solutions for a range of surveillance applications. It is anticipated that additional research in these areas would improve computer vision systems’ capacity for security and surveillance.

## Proposed methodology

**Fig. 1:**
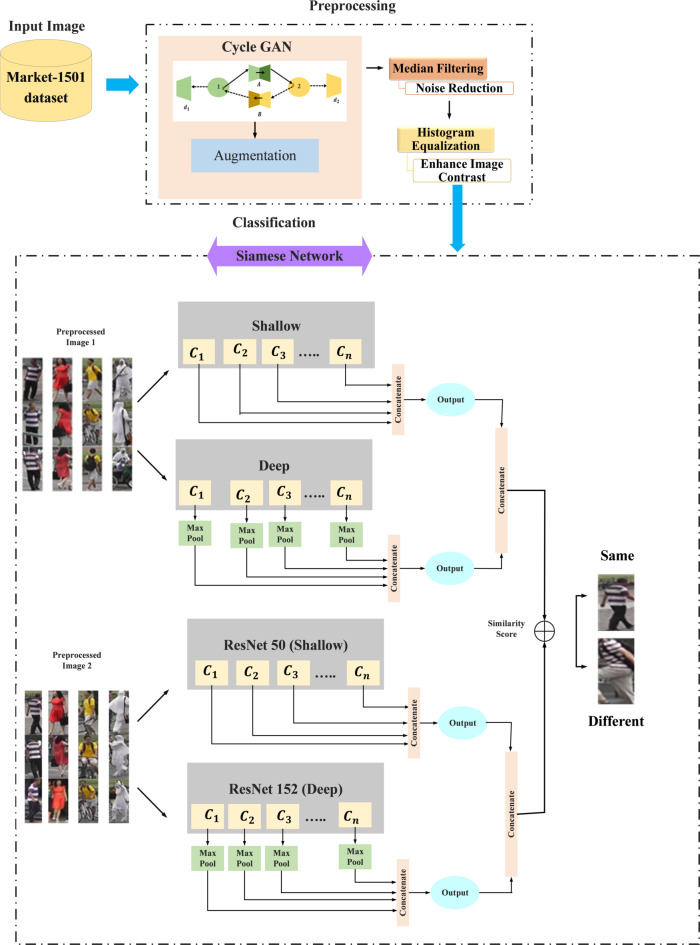
Overall architecture of the proposed methodology.

Inter-camera person re-ID, which enables recognition of humans across various camera perspectives, is an essential procedure in surveillance systems. It addresses issues that are critical for security applications, such as lighting variations, viewpoint shifts, and occlusions. In this context, improving single-shot unsupervised domain adaptation addresses issues with illumination, viewpoint, and occlusion while also improving the identification process without labeled data. This method improves person re-identification system reliability by improving domain adaptation approaches. Data collection, preprocessing (augmentation, median filtering, histogram equalization), and classification (using Siamese networks, Conv50, and Conv152) are all included in the technique. The general design of the suggested methodology is depicted in Fig. 1.

### Preprocessing

The dataset used for experiments is Market 1501 dataset^25^. Preprocessing stage is an essential part of the entire data processing flow. Its main objective is to modify and improve images so they are prepared for further analysis or training. Stereo and depth-based imaging approaches have been shown to improve human monitoring under varying environmental conditions, and this motivates the robust preprocessing design^26,27^.In this work, CycleGAN is used for data augmentation and style adaptation, followed by median filtering for noise reduction and histogram equalization to enhance image contrast. These steps improve the visibility of discriminative features and increase the robustness and generalization capability of the proposed Siamese network for person re-identification.

#### Data augmentation using Cycle-Consistent generative adversarial network (Cycle GAN)

**Fig. 2:**
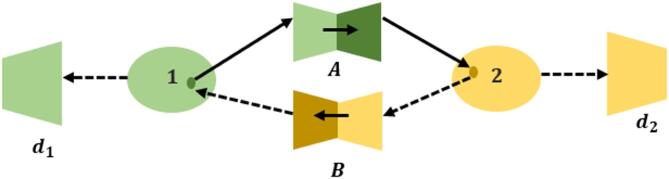
Cycle GAN architecture.

Data augmentation in CycleGAN refers to the generation of new images in a training set by translating the image from one domain to another, keeping its semantics and information. This method increases the variety of dataset data and is particularly helpful when translating one image to another in computer vision and machine learning applications. It includes editing existing data samples in various ways, for example, to generate new images with different features that are important while still preserving relevant features. Some common methods include rotation, flipping, noise reduction, scaling, zooming, and color contrast. The Inter-camera Person Re-ID framework mimics the real-world variations among multiple cameras by modifying viewpoint, illumination, background, and image quality adjustments to enrich the dataset. The approach proposed in this paper shows effective reflection of the problems in multi-camera surveillance scenarios, contributing to the development of more robust and generalizable machine learning models for improving the accuracy and reliability of human re-identification systems under real-world situations. CycleGAN is chosen because it enables cross-domain style transfer between different camera views (e.g., Camera A to Camera B) without requiring paired images It explicitly learns the visual style differences between domains, including illumination, background, and color distribution, in contrast to simple augmentation techniques. By lowering inter-camera domain shift, this capability directly tackles the fundamental problem of unsupervised domain adaptation. This approach contributes to developing domain-invariant representations for person re-identification tasks, where images are normalized in terms of the appearance of people on cameras, hence promoting higher accuracy and robustness in person re-identification systems in surveillance scenarios. Figure 2 shows the Cycle GAN architecture.

Two generators, $\:A\:and\:B$, and two adversarial discriminators, $\:{d}_{1}$ and $\:{d}_{2}$, are components of the CycleGAN model. $\:A$ is required by $\:{d}_{2}$ to convert images from $\:1$ into outputs that are identical to those of domain $\:2$, and vice versa for $\:{d}_{1}$ and $\:B$.

CycleGAN provides two generator-discriminator pairs, ($\:A,\:{d}_{t}$) and ($\:B,\:{d}_{s}$), which maps a sample from the source (target) domain to the target (source) domain, producing a sample that is indistinguishable from those in the target (source) domain. For generator $\:A$ and its associated discriminator $\:{d}_{t}$, the adversarial loss is in Eq. (1)

1 $$\:{\mathcal{L}}_{tadv}\left(A,\:{d}_{t},\:{p}_{x},\:{p}_{y}\right)={E}_{y\sim{p}_{y}}\left[{\left({d}_{t}\left(y\right)-1\right)}^{2}\right]+{E}_{x\sim{p}_{x}}\left[{\left({d}_{t}\left(x\right)\right)}^{2}\right]$$

where $\:{p}_{x}$ and $\:{p}_{y}$ represent sample distributions in the source and target domains, respectively. The adversarial loss for generator $\:B$ and its related discriminator $\:{d}_{s}$ is in Eq. (2)

2 $$\:{\mathcal{L}}_{sadv}\left(B,\:{d}_{s},\:{p}_{y},\:{p}_{z}\right)={E}_{x\sim{p}_{x}}\left[{\left({d}_{s}\left(x\right)-1\right)}^{2}\right]+{E}_{y\sim{p}_{y}}\left[{\left({d}_{s}\left(B\left(y\right)\right)\right)}^{2}\right]$$

CycleGAN uses a cycle-consistent loss to reduce the number of possible mapping functions due to a lack of paired training data. The loss aims to recover the original image after each cycle of translation and reverse translation. The loss is consistent with the cycle in Eq. (3)

3 $$\:{\mathcal{L}}_{tadv}\left(A,B\right)={E}_{x\sim{p}_{x}}\left[{\parallel{B}\left(A\left(x\right)\right)-x\parallel}_{1}\right]+{E}_{y\sim{p}_{y}}\left[{\parallel{A}\left(B\left(y\right)\right)-y\parallel}_{1}\right]$$

In addition to cycle-consistent loss and adversarial loss, employ the target domain identity constraint as a translation auxiliary for images. To regularize the generator to be the identity matrix on samples from the target domain, the target domain identity constraint is imposed. It is expressed as in Eq. (4)

4 $$\:{\mathcal{L}}_{ide}\left(A,B,{p}_{x},\:{p}_{y}\right)={E}_{x\sim{p}_{x}}\left[{\parallel{B}\left(x\right)-x\parallel}_{1}\right]+{E}_{y\sim{p}_{y}}\left[{\parallel{A}\left(y\right)-y\parallel}_{1}\right]$$

**Fig. 3:**
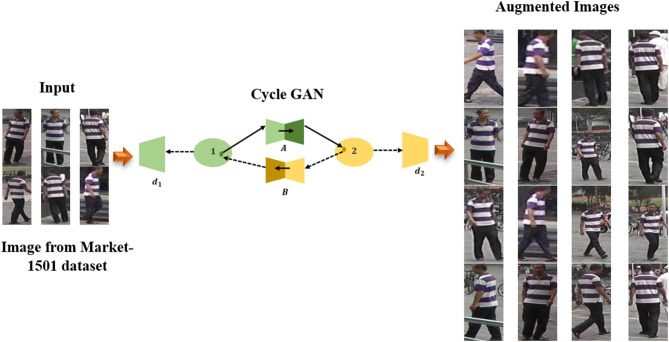
Augmentation process.

Input images may undergo color changes by generators $\:A\:and\:B$ in the absence of $\:{\mathcal{L}}_{ide}$. Figure 3 demonstrates the Augmentation process.

#### Noise reduction using median filter

Following Cycle GAN augmentation, the enhanced images are subjected to Median Filtering to remove noises resulting from noise reduction. A nonlinear digital filtering method called the median filter is essential for eliminating noise from signals or images. It is a preprocessing step that is necessary to provide better processing results, including edge detection. In image preprocessing, the median filter is essential for noise reduction in the framework of inter-camera person reidentification. Median filtering is a commonly used approach in digital image processing and is crucial for both image and signal processing applications because it may keep edges while lowering noise. It is a vital tool in workflows involving data preparation and augmentation because of its computing efficiency and resilience against outlier values.

#### Enhancing image contrast using histogram equalization

It is a key method for increasing visual contrast by exploiting the histogram of the filtered image. HE is very useful for scientific photos like thermal, satellite, or x-ray images; in fact, these are the same kinds of images that are often applied with fake color, even though it is often employed to produce surreal effects in photographs. Furthermore, histogram equalization may have undesirable effects (such as pronounced image gradients) when applied to photographs with low color depth. If it is applied to an 8-bit image that is presented using an 8-bit grayscale palette, for example, it will further reduce the image’s color depth, or the number of different shades of gray. photos with a far higher color depth than palette sizes, such as continuous data or 16-bit grayscale photos, yield the greatest results from histogram equalization. In image processing, histogram equalization is a method for redistributing pixel intensities to improve contrast. Histogram equalization can be used in the context of Inter-camera Person re-ID to enhance the uniformity of visual features between several cameras. It can lessen differences in illumination, camera settings, and image quality between various camera viewpoints by modifying the pixel intensity distribution. To enable more accurate and reliable person re-identification in surveillance systems across numerous camera networks, this preprocessing phase aims to standardize the appearance of humans captured by various cameras. Examine a digital picture with a probability distribution and gray levels between [0, L − 1. Equation (5) can be used to ascertain the picture’s function. (5)

5 $$\:P\left({r}_{k}\right)=\frac{{n}_{k}}{N}\:\:\:\:\:\:\:\:\:\:k=0,\dots\:,L-1$$

Where $\:{n}_{k}$, number of pixels in the image with a gray level; $\:{r}_{k}$, $\:kth$ gray level. Another way to compute the Cumulative Distribution Function (CDF) is as follows in Eq. (6)

6 $$\:C\left({r}_{k}\right)=\sum\:_{i=0}^{i=k}P\left({r}_{i}\right)\:\:\:\:k=0,\dots\:,L-1,\:0\underset{\_}{<}C\left({r}_{k}\right)\underset{\_}{<}1\:\:\:\:\:\:\:\:$$

Equation (6) is used by HE to adjust the gray level $\:{S}_{k}$ to gray level $\:{r}_{k}$ of the input image is expressed in Eq. (7)

7 $$\:{S}_{k}=\left(L-1\right)\times\:C\left({r}_{k}\right)$$

The Gray Level $\:{S}_{k}The$ variations can be calculated using the standard histogram equalization technique in Eq. (8)

8 $$\:{\varDelta\:S}_{k}=\left(L-1\right)\times\:P\left({r}_{k}\right)$$

Equation (8) indicates that the PDF of the input image at gray level $\:{r}_{k}$ has a direct relationship with the distance amid $\:{S}_{k}$ and $\:{S}_{k}+1$. Equation (6)’s quantization operation and summarizing characteristics produce undesirable effects of the standard histogram equalization approach (HE).

### Classification using Siamese network

Siamese neural networks, sometimes referred to as twin neural networks, are a particular kind of artificial neural network designed to perform tasks involving inter-camera person re-identification. This model operates by processing two distinct input vectors simultaneously using identical weights to generate similar output vectors. Often, one of the output vectors is predetermined to establish a benchmark for evaluating the other output vector. This setup draws parallels to contrasting unique signatures in the context of human reidentification across varying cameras, albeit functioning as a distance function for locality-sensitive hashing. The utilization of Siamese networks is extensive, particularly in scenarios like facial recognition within surveillance images, matching queries with indexed documents, and identifying individuals from different camera perspectives. These networks play a vital part in the functionality of robust and precise person re-ID systems due to their effectiveness in tasks such as facial recognition and verification, which involve comparing existing images with live camera feeds to authenticate or recognize individuals. The initial processing involves Image 1 and Image 2 captured by cameras 1 to 6, proceeding to the shallow Conv50 and deep Conv152 layers. The recommendation is to extract features from two layers shallow and deep selected according to their receptive fields, as opposed to focusing just on extracting intermediate and deep feature maps. These two backbone layers are chosen based on the receptive field, which represents the region of the input space that a CNN feature scans. A 3 × 3 output feature map is created by applying a convolution with a 3 × 3 kernel, 1 × 1 padding, and 2 × 2 stride on a 5 × 5 input picture. These feature maps are then concatenated before moving on to the layers that flatten. Repeating the convolution process leads to a final 1 × 1 output feature map.

**Fig. 4:**
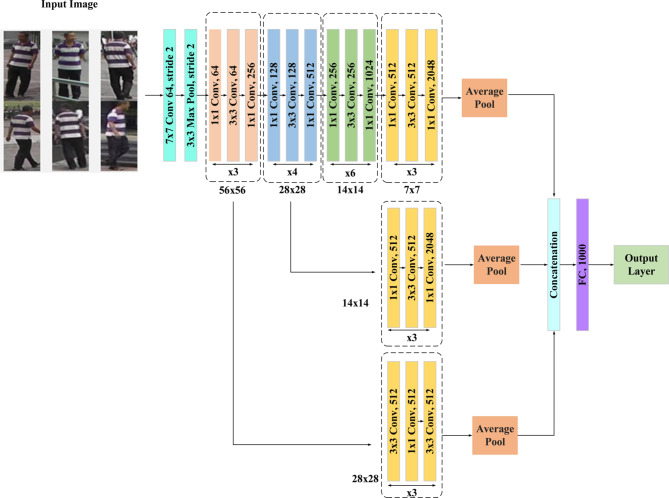
ResNet 50 architecture.

Figure 4 shows the ResNet 50 architecture. A type of convolutional neural network (CNN) called a ResNet (Residual Network) architecture. ResNet-50 adds residual connections to solve the disappearing gradient issue and makes training deeper networks easier than with conventional CNNs like VGGNet, which employ a sequential stacking of convolutional layers. The ideas of the ResNet-50 (shallow) design are modified for inter-camera person re-identification to accommodate its intricate nature. With 50 levels, ResNet-50, in comparison to deeper designs such as ResNet-152 or ResNet-101, which have 152 and 101 layers, respectively, ResNet-50 is considered relatively shallow. Though shallow in contrast, ResNet-50 is a robust and well-liked architecture for many computer vision applications, including object detection, picture classification, and person re-ID. Through observance to certain architectural guidelines, the architecture preserves a simple but effective structure. First off, each layer has the same number of filters, which are all based on the size of the output feature map that is intended. Second, ResNet-50 doubles the number of filters to preserve computational efficiency across layers to account for the halves of feature map sizes. ResNet’s main innovation is the addition of “bottleneck” or residual blocks. These building pieces guarantee that the network can efficiently learn residual mappings. Two fundamental design ideas are followed by each bottleneck block: (i) keeping the number of filters constant for output feature map sizes, and (ii) modifying the number of filters when feature map sizes are half to maintain computational complexity. With convolutional layers and a stride of 2 × 2, down-sampling a critical operation in inter-camera person re-ID tasks is accomplished. Batch normalization is performed both before and after each convolutional layer of the Rectified Linear Unit (ReLU) activation function to offer stable and efficient training. When the input and output dimensions are the same, an identity shortcut is utilized; when dimensions increase, a projection shortcut employing 1 × 1 convolutions is used. By ensuring that gradient information may flow efficiently during training, these shortcuts enable deep network optimization to be accomplished. The design and ideas of ResNet-50 enable the development of dependable and accurate models that can manage the challenges posed by disparate camera viewpoints, lighting scenarios, and image quality in the context of inter-camera person re-identification. The network’s depth, efficiency in parameter learning, and smart shortcut connections enable it to learn discriminative characteristics for human re-identification tasks across several camera views.

**Fig. 5:**
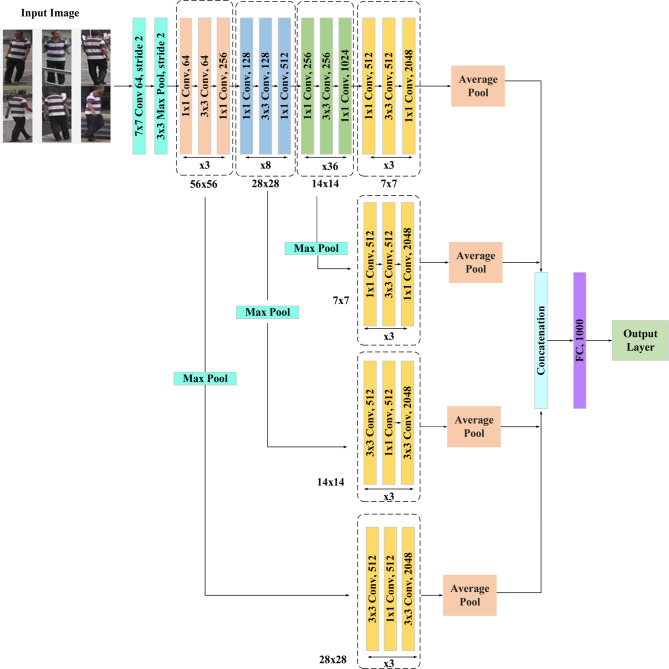
ResNet-152 architecture.

Figure 5 exhibits the ResNet-152 architecture. One deep CNN that is relevant to inter-camera person re-ID is ResNet-152. Within the field of re-identification, ResNet-152 is essential for extracting highly discriminative characteristics from human images taken with several cameras. Because of its depth, ResNet-152 can accurately reidentify people from a variety of camera perspectives by capturing minute characteristics and appearance differences including clothing, posture, and lighting. ResNet 152 features a residual learning unit to shield deep neural networks against abuses. A shortcut link on this unit makes it possible to add further inputs and outputs. It is constructed as a feed-forward network. The main benefit is that it raises classification accuracy without making the model more complex. Additional three-layer blocks are combined to create the ResNet − 152 layer. The architecture of the 152-layer ResNet is simpler than that of the 34-layer VGG-16/19 network. The relationships between the residual blocks were very beneficial to the residual connections in the ResNet architecture. It improves model development by increasing the network’s capacity while preserving the information acquired during training. A deep CNN design with a residual network is the Conv 152 layer. The number “152” in ResNet-152 indicates how many convolutional, pooling, and fully connected layers there are in the network. Because of its many layers, which enable it to recognize complex patterns and characteristics in images during training, it is referred to as having a “deep” architecture. In computer vision, deeper networks like ResNet-152 are frequently employed for challenging tasks like segmentation, object detection, and image recognition, where precise and significant predictions depend on high-level feature representations.

**Fig. 6:**
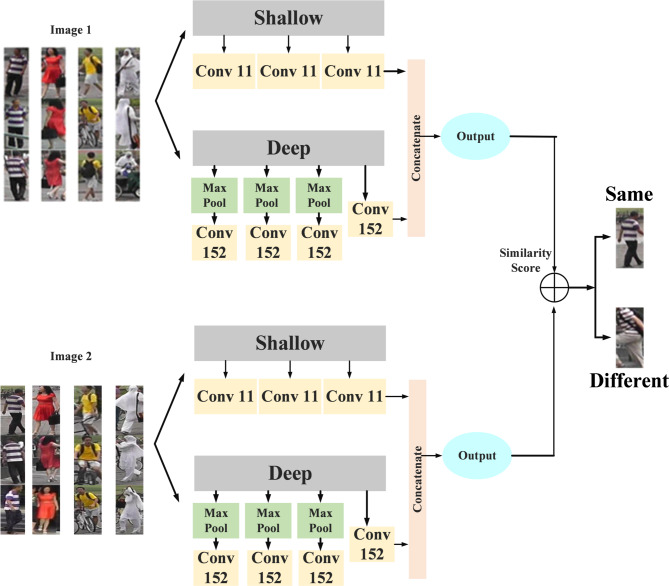
Siamese Network.

Figure 6 depicts the architecture of the Siamese Network. The architecture demonstrates the processing of two pre-processed images, Image 1 and Image 2, by six cameras functioning as input. After these two images go through processing with two distinct CNN: Conv50 (ResNet-50) and Conv152 (ResNet-152). Conv152 (ResNet-152) operates on the inputs from both Image 1 and Image 2 before the max pooling layer is applied to each, hence producing outputs. The resultant of the operation of Conv50 (ResNet-50) and Conv152 (ResNet-152) is combined through concatenation. Concatenation means to merge two or more arrays along a specified axis. The merged output is further input into another CNN, which outputs a similarity score. This score represents the similarity between the two input images. In basically, the system receives two pictures as input and generates a similarity score between them. This score can be used to figure out the effects of a search. Finally, the output signifies whether the images are the same or different.

## Result and discussion

The results and a discussion of the suggested model are provided in this section. This method improves person re-identification system reliability by improving domain adaptation approaches. Data collection, preprocessing (augmentation, median filtering, histogram equalization), and classification (using Siamese networks, Conv50, and Conv152) are all included in the technique. The recently developed framework is compared to other models, including Proposed, ST-MC^16^, PPAN^20^, and AdaDC^21^, to evaluate how significantly its performance has improved.

## Evaluation setup

The recommended framework has been implemented in the PYTHON platform. The suggested framework has been evaluated utilizing the Market-1501 dataset. The dataset consists of 12,936 images in the training set and 19,732 images in the test set. From the training set, a 70/30 split was applied to create the final training and validation subsets. Accordingly, 9,055 images (70% of 12,936) were used for training, and 3,881 images (30% of 12,936) were used for validation, while all 19,732 images were kept exclusively for testing.

## Performance metrics

Several Metrices like Accuracy, Precision, F-Score, Specificity, Sensitivity, MCC, NPV, MSE, MAE, NMSE, RMSE, and MAP are used for performance evaluation.

Ablation Study:

**Table 2:** Split data 70/30 for performance metrics with cycle GAN.

Model	ST-MC^16^	PPAN^20^	AdaDC^21^	Proposed
Accuracy	95.124	94.408	94.791	98.124
Precision	95.856	94.008	95.085	98.856
F-score	95.467	94.889	95.072	97.667
Specificity	95.201	94.065	95.083	98.201
Sensitivity	95.014	95.013	95.0194	98.014
MCC	95.856	95.862	95.0842	97.816
NPV	95.182	95.781	95.543	97.062
MSE	0.34206	0.288242	0.304156	0.26885
MAE	0.34542	0.308762	0.31541	0.27964
NMSE	0.305773	0.31542	0.31745	0.28753
RMSE	0.291074	0.30185	0.31547	0.25158

Table 2 offers a thorough analysis of performance metrics amongst various models when it comes to human re-identification between cameras. The table is organized into rows that correspond to distinct evaluation metrics, while the columns reflect several models, such as the ST-MC, PPAN, AdaDC, and the proposed model. Notably, the Proposed model performs exceptionally well on several criteria. It obtained 98.124% accuracy, 98.856% precision, 97.667% F-score, 98.201% specificity, 98.014% sensitivity, 97.816% MCC, 97.062% NPV, 0.26885 MSE, 0.27964 MAE, 0.28753 NMSE, RMSE, and 0.29874 MAPE. Together, these measures show how the Proposed model outperforms the other models in the comparison in terms of resilience and accuracy in correctly recognizing and re-identifying individuals across various camera views.

**Table 3:** Split data 70/30 for performance metrics with cycle GAN.

Model	ST-MC^17^	PPAN^21^	AdaDC^22^	Proposed
Accuracy	96.644	95.648	95.991	99.268
Precision	96.759	95.508	95.885	99.866
F-score	96.367	95.189	95.852	99.567
Specificity	96.631	95.735	95.789	98.951
Sensitivity	96.109	96.197	96.5194	98.894
MCC	96.556	96.062	96.4842	99.116
NPV	96.832	96.881	95.543	99.543
MSE	0.33506	0.276242	0.293256	0.25885
MAE	0.33542	0.297752	0.30631	0.26964
NMSE	0.290773	0.30442	0.30655	0.27753
RMSE	0.281074	0.29861	0.30446	0.24158
MAPE	0.300273	0.30681	0.31615	0.28874

For inter-camera person re-identification, Table 3 provides a thorough comparison of performance metrics for several models, including the Proposed model, PPAN, AdaDC, and ST-MC. Every parameter sheds light on how well the algorithms perform at reliably identifying people from various camera angles. At 99.268% accuracy, 99.866% precision, 99.567% F-score, 98.951% specificity, 98.894% sensitivity, 99.116% MCC, 99.543% NPV, 0.25885 MSE, 0.26964 MAE, 0.27753 NMSE, RMSE, and 0.28874 MAPE, the Proposed model performs exceptionally well by all measures.

**Fig. 7:**
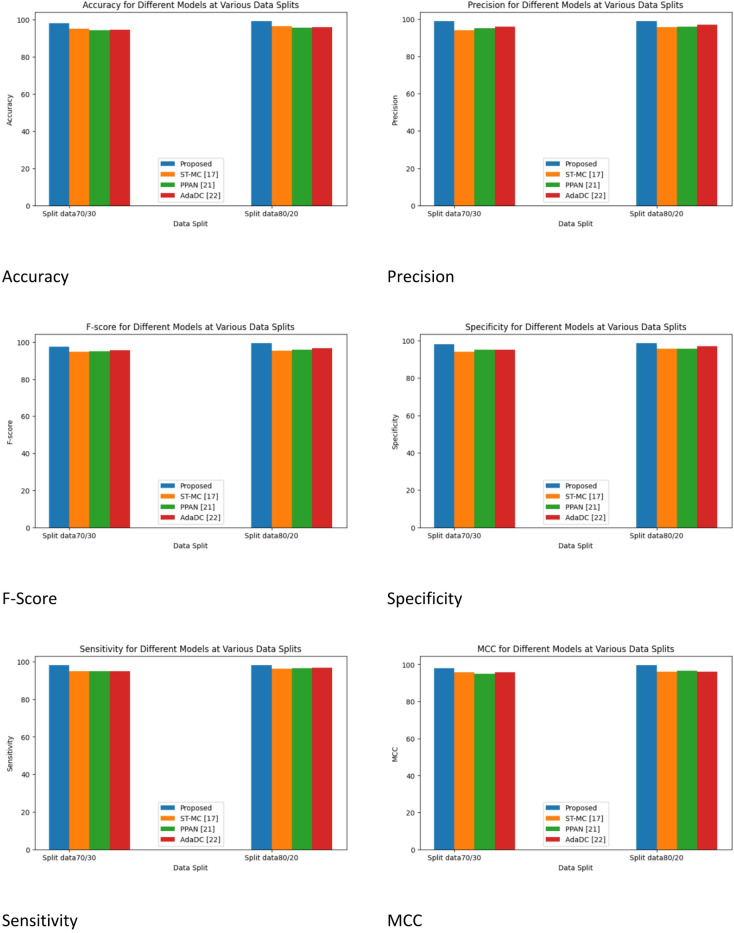

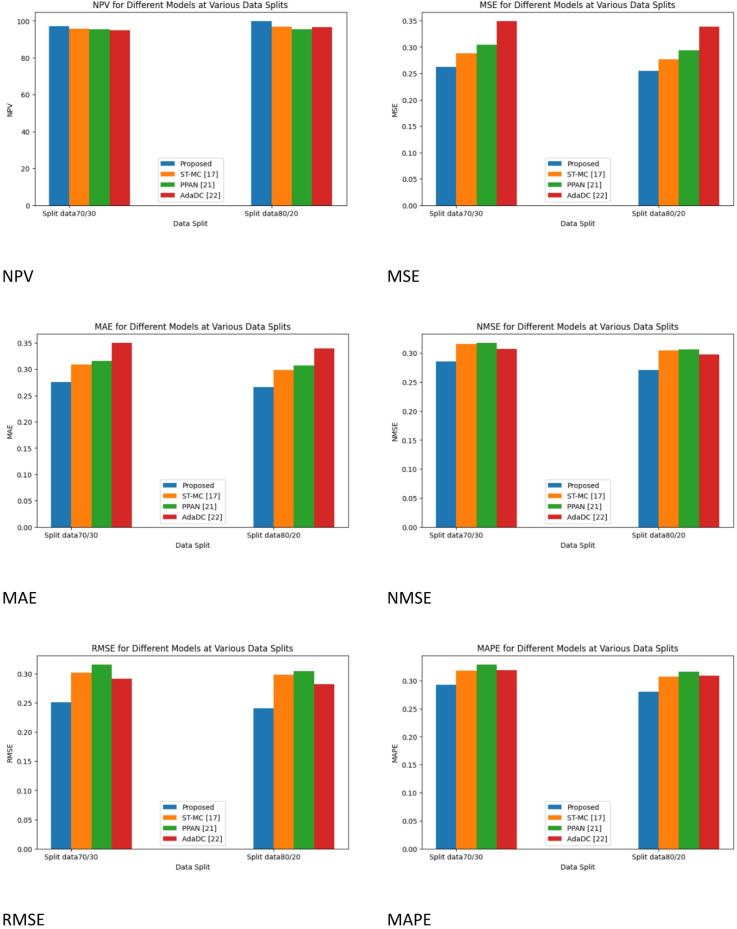
Performance metrics for with Cycle GAN.

Figure 7 demonstrates the comparative analysis of the performance metrics of the existing works with Cycle GAN. Performance metrics such as Accuracy, Precision, F-Score, Specificity, Sensitivity, MCC, NPV, MSE, MAE, NMSE, RMSE, and MAPE are compared with different methods like ST-MC^16^, PPAN^20^, and AdaDC^21^ and proposed.

**Table 4:** Split data 70/30 for performance metrics without cycle GAN.

Model	ST-MC^17^	PPAN^21^	AdaDC^22^	Proposed
Accuracy	92.334	93.449	92.996	96.044
Precision	92.552	93.972	93.864	96.466
F-score	93.661	93.969	94.908	96.667
Specificity	92.226	94.661	93.383	96.201
Sensitivity	93.329	93.209	94.0194	96.914
MCC	92.871	93.68	92.0842	95.115
NPV	93.885	93.779	92.543	95.622
MSE	0.36302	0.308242	0.324156	0.28695
MAE	0.36442	0.338762	0.33941	0.29064
NMSE	0.365773	0.34542	0.33745	0.30862
RMSE	0.321074	0.32885	0.32967	0.27546
MAPE	0.332776	0.33446	0.34647	0.31774

With a 70/30 data split, Table 4 compares the performance metrics of various models for inter-camera person re-identification, without the Cycle GAN technique. The accuracy values indicate the overall correctness of the models and range from 92.334% to 96.044%. The precision varies from 92.552% to 96.466%, with higher numbers denoting better precision and the avoidance of false positives. F-score values range from 93.661% to 96.667%, indicating a balance between sensitivity and precision. The range of specificity, which denotes accurate negative identifications, is 92.226% to 96.201%, suggesting different degrees of false alarms. The model’s sensitivity, which varies from 93.209% to 96.914%, shows how well it can detect positives. The MCC values give an overall measure of categorization performance, ranging from 92.084% to 95.115%. Table 5 reports the performance metrics based on an 80/20 data split without the use of CycleGAN.

**Table 5:** Split data 80/20 for performance metrics without cycle GAN.

Model	ST-MC^17^	PPAN^21^	AdaDC^22^	Proposed
Accuracy	93.534	94.448	93.991	96.668
Precision	93.551	93.608	93.885	96.866
F-score	94.662	94.189	94.998	96.867
Specificity	93.931	94.735	94.789	96.951
Sensitivity	94.009	94.897	94.642	96.894
MCC	94.986	93.989	94.8842	95.916
NPV	94.339	93.881	93.543	95.943
MSE	0.35506	0.296242	0.313256	0.27885
MAE	0.34542	0.327052	0.32631	0.28964
NMSE	0.340673	0.33922	0.32655	0.29753
RMSE	0.303064	0.31861	0.31846	0.26158
MAPE	0.310076	0.32681	0.33615	0.30874

Without the Cycle GAN method, the table shows performance numbers for several models in inter-camera person re-identification using an 80/20 data split. The accuracy results exhibit a range of 93.534% to 96.668%, which indicates the models’ overall accuracy in distinguishing humans from different cameras. The precision scores fall between 93.551% and 96.866%, which represents the models’ ability to prevent erroneous positives. The F-score falls between 94.662% and 96.867%, representing a compromise between sensitivity and precision. The range of specificity values, which suggest accurate negative identifications, is 93.931% to 96.951%. Sensitivity values, which show how well the models detect positives, vary from 94.009% to 96.894%. The classification performance is measured by the MCC values, which vary from 93.989% to 95.916%.

**Fig. 8:**
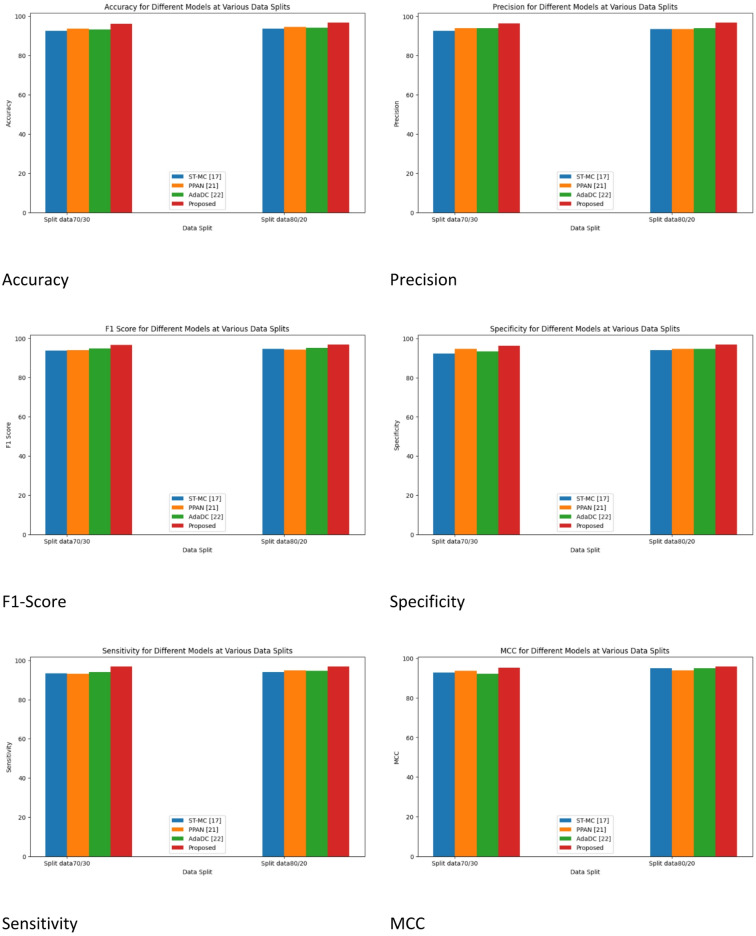

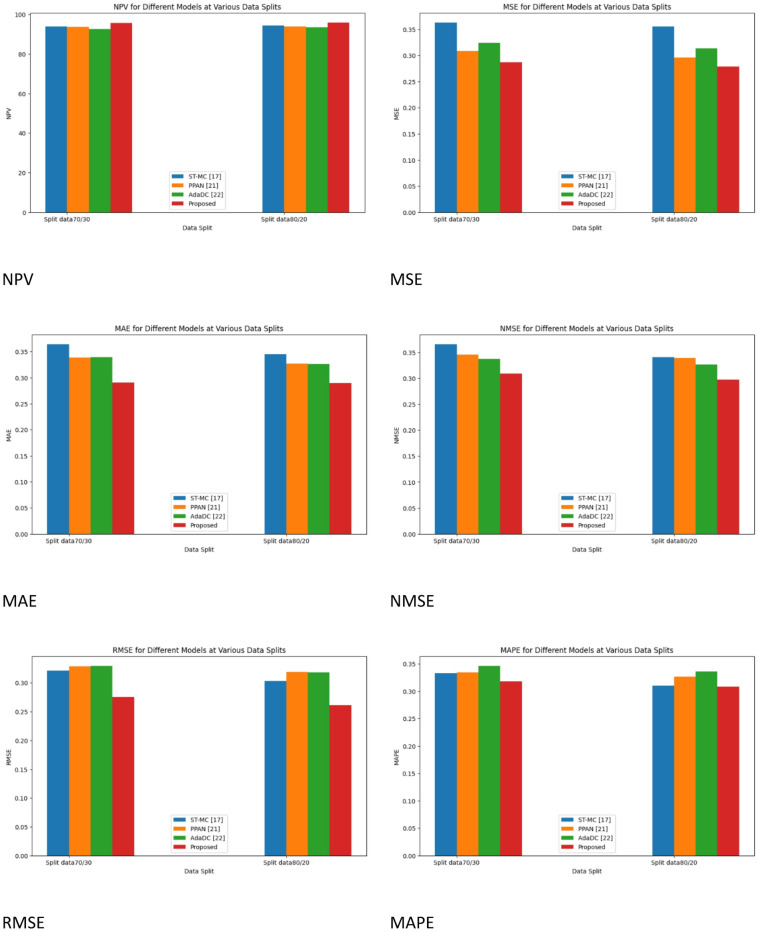
Performance metrics for without Cycle GAN.

Figure 8 demonstrates the comparative analysis of the performance metrics of the existing works without Cycle GAN.

## Overall performance analysis for augmentation

Augmentation is utilized in machine learning and computer vision to diversify training datasets by generating new data factors through random modifications. It includes rotation, scaling, cropping, flipping, and adding noise. Data augmentation helps prevent overfitting by exposing models to a wider variety of data versions, improving generalization and robustness. Models can perform better with unseen data by artificially expanding the dataset, making data augmentation a valuable tool in enhancing machine learning model performance.

**Table 6:** Split data 70/30 for performance metrics for Augmentation.

Model	ST-MC^17^	PPAN^21^	AdaDC^22^	Proposed
Accuracy	91.644	92.449	91.996	95.044
Precision	91.622	93.972	91.864	95.466
F-score	92.641	93.969	91.908	95.067
Specificity	92.626	94.661	91.733	95.006
Sensitivity	92.029	93.209	91.1144	95.914
MCC	91.871	93.68	91.8842	95.115
NPV	92.885	93.779	91.443	95.622
MSE	0.35302	0.308242	0.334156	0.28695
MAE	0.35442	0.338762	0.34941	0.29064
NMSE	0.355773	0.34542	0.38745	0.30862
RMSE	0.311774	0.32885	0.33967	0.27546
MAPE	0.329076	0.33446	0.35668	0.31774

Performance metrics for several inter-camera person re-identification models are shown in Table 6. Training and testing data are divided 70/30 for data augmentation strategies. Between 91.644% and 95.044% is the accuracy range, with the suggested model having the maximum accuracy. Between 91.622% and 95.466% are the precision values; the PPAN model has the highest precision. The F-score shows that the performance of the models is balanced, ranging from 91.908% to 95.067%. MCC values, specificity, and sensitivity show how well the suggested model is in comparison to other models. Furthermore, measures such as MSE, MAE, NMSE, RMSE, and MAPE offer valuable information about the predicted errors and deviations of the models from real values; lower values signify superior performance.

**Table 7:** Split data 80/20 for performance metrics for Augmentation.

Model	ST-MC^16^	PPAN^20^	AdaDC^21^	Proposed
Accuracy	92.102	93.448	92.991	95.316
Precision	93.051	94.008	92.885	95.806
F-score	93.262	94.009	92.998	95.661
Specificity	93.306	94.735	92.789	95.554
Sensitivity	93.339	94.897	92.642	96.018
MCC	92.076	93.989	92.8842	95.916
NPV	93.339	93.881	92.543	95.943
MSE	0.34006	0.290042	0.322256	0.27625
MAE	0.34442	0.320052	0.33631	0.28004
NMSE	0.340673	0.33065	0.33655	0.29503
RMSE	0.306064	0.32361	0.32846	0.26006
MAPE	0.300076	0.32883	0.32615	0.30703

Performance metrics for several models in inter-camera person re-identification are shown in Table 7. Data augmentation is included and an 80/20 data split is used for training and testing. Between 92.102% and 95.316% is the accuracy range, with the suggested model having the maximum accuracy. 92.885% to 95.806% is the range of precision values, with the PPAN model exhibiting the highest precision. An F-score of 92.998% to 95.661% indicates that the performance of the models is balanced. MCC values, specificity, and sensitivity show how well the suggested model is in comparison to other models. Furthermore, measures such as MSE, MAE, NMSE, RMSE, and MAPE offer valuable information about the predicted errors and deviations of the models from real values; lower values signify superior performance.

## Overall performance analysis for traditional Siamese network

**Table 8:** Split data 70/30 for performance metrics for traditional Siamese Network.

Model	ST-MC^16^	PPAN^20^	AdaDC^21^	Proposed
Accuracy	93.644	92.449	92.996	94.944
Precision	93.622	93.972	92.864	95.866
F-score	93.991	93.969	92.908	95.967
Specificity	93.229	94.661	92.733	95.696
Sensitivity	93.669	93.209	92.1144	95.895
MCC	93.706	93.68	92.8442	95.695
NPV	94.885	93.779	92.893	95.962
MSE	0.36302	0.308242	0.344156	0.30995
MAE	0.36442	0.338762	0.36941	0.32064
NMSE	0.365773	0.34542	0.39745	0.33862
RMSE	0.338774	0.32885	0.33989	0.30546
MAPE	0.339076	0.33446	0.35088	0.32774

Performance metrics for conventional Siamese network models in inter-camera person re-identification are shown in Table 8, with training and testing data divided 70/30. Between the compared models, the ST-MC model achieves the maximum accuracy, with an accuracy ranging from 92.449% to 94.944%. The models’ precision values, which range from 92.864% to 95.866%, show how well they can prevent false positives. The F-score, which displays balanced performance across models and reflects the harmonic mean of precision and recall, ranges from 92.908% to 95.967%. The assessment of model efficacy also takes into account the values of specificity, sensitivity, and MCC; higher values often indicate greater performance in properly detecting positive and negative cases. Lower values indicate better model accuracy and reliability. Metrics such as MSE, MAE, NMSE, RMSE, and MAPE offer insights into prediction errors and disparities between actual and anticipated values.

**Table 9:** Split data 80/20 for performance metrics for traditional Siamese Network.

Model	ST-MC^16^	PPAN^20^	AdaDC^21^	Proposed
Accuracy	94.102	93.898	92.701	95.116
Precision	94.051	93.008	92.885	95.003
F-score	94.262	93.009	92.998	96.701
Specificity	94.306	93.735	92.789	96.244
Sensitivity	94.339	93.897	92.642	96.018
MCC	93.076	93.992	92.8842	96.916
NPV	94.339	93.881	92.543	96.043
MSE	0.35006	0.292542	0.322256	0.29625
MAE	0.35442	0.326505	0.33631	0.31004
NMSE	0.350673	0.33965	0.33655	0.33503
RMSE	0.326064	0.31668	0.32846	0.29006
MAPE	0.320076	0.32933	0.32615	0.30703

Based on an 80/20 split for training and testing data, Table 9 shows performance metrics for conventional Siamese network models in inter-camera person re-identification. Between the compared models, the suggested model achieves the maximum accuracy, with accuracy values ranging from 92.701% to 95.116%. The models’ precision values, which range from 92.885% to 95.003%, show how well they can prevent false positives. The F-score, which reflects equal performance among models in terms of precision and recall, ranges from 92.998% to 96.701%. Values for specificity, sensitivity, MCC, and NPV shed light on how well the models distinguish between positive and negative occurrences. The efficacy of conventional Siamese network models in inter-camera person re-identification tasks is demonstrated by lower values of MSE, MAE, NMSE, RMSE, and MAPE, which signify improved model accuracy and reliability, especially when an 80/20 data split is used.

**Fig. 9:**
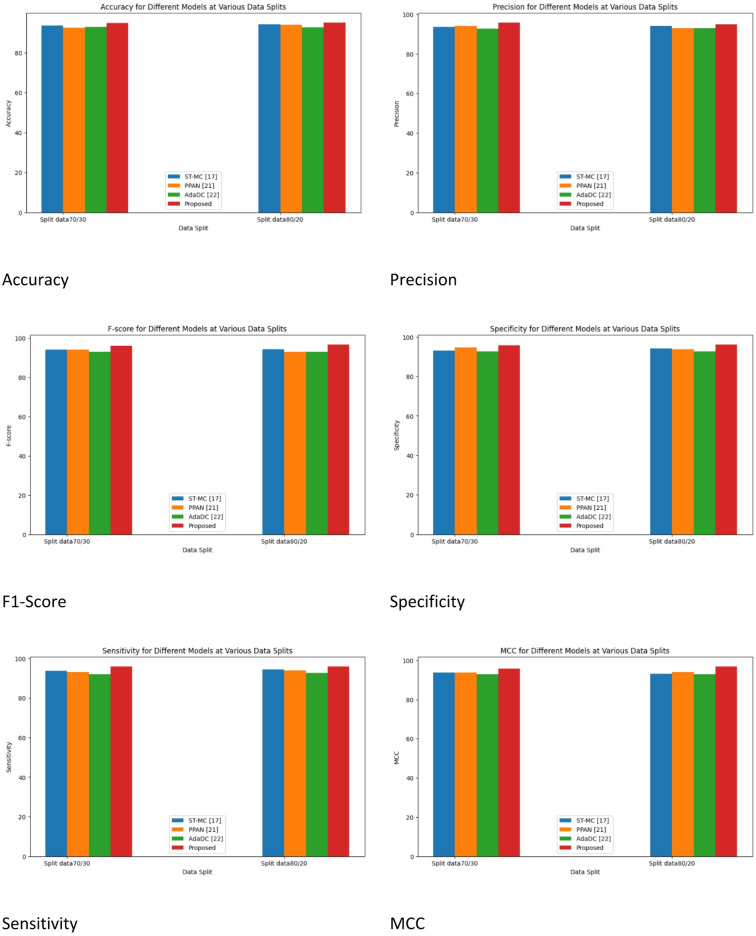

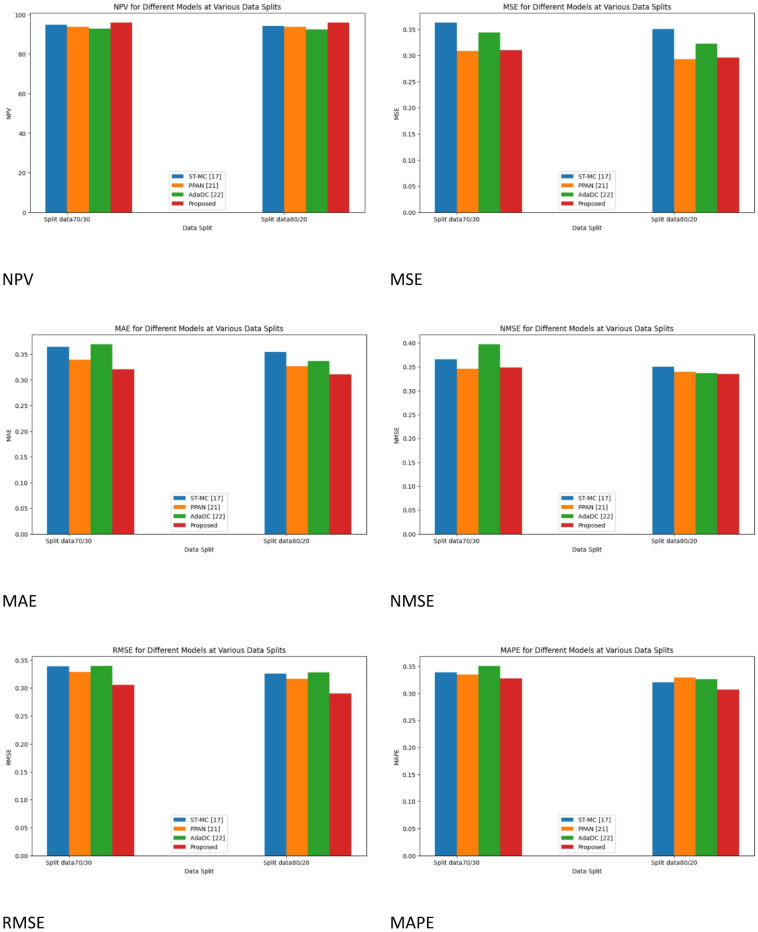
Performance Metrics for Traditional Siamese Network.

Figure 9 demonstrates the comparative analysis of the performance metrics of the existing works for the Traditional Siamese Network.

## Conclusion

An improved Single Shot Unsupervised Domain Adaptation (SSUDA) framework for inter-camera person re-identification was presented in this work. It was intended to handle practical issues like viewpoint variation, illumination changes, background clutter, and partial occlusions. A Siamese network architecture with ResNet-50 and ResNet-152 as backbone feature extractors was combined with a thorough preprocessing pipeline that included CycleGAN-based augmentation, median filtering for noise reduction, and histogram equalization for contrast enhancement. With accuracy ranging from 92.701% to 95.116% and consistently high precision, F-score, sensitivity, and MCC values, experimental results based on an 80/20 training–testing split show that the suggested model outperformed traditional Siamese models. The robustness and dependability of the suggested method in intricate surveillance settings are further supported by the decreased error metrics (MSE, RMSE, MAE, NMSE, and MAPE).

These results show that inter-camera person re-identification performance in safety and monitoring applications can be greatly enhanced by the suggested SSUDA-based framework. In order to improve accuracy in dynamic environments, future work will concentrate on expanding the model to large-scale cross-dataset evaluation, optimizing real-time implementation in embedded surveillance systems, and adding temporal and multi-modal features.

**Appendix 1**.

**Table:** 

Accuracy	$\:Accuracy=\frac{TP+TN}{TP+FP+FN+TN}$
Precision	$\:Precision=\frac{TP}{FP+TP}$
F-Score	$\:F-Measure=\frac{Presision.\:Recall}{Presision+\:Recall}$
Recall	$\:Recall=\frac{TP}{TP+FN}$
Specificity	$\:Specificity\:=\frac{TN}{FP+TN}$
Sensitivity	$\:Sensitivity=\frac{TP}{TP+FN}$
Matthews Correlation Coefficient (MCC)	$\:MCC=\frac{\left(TP\mathrm{*}TN\right)-\left(FP\mathrm{*}FN\right)}{\sqrt{(TP+FP)(TP+FN)(FP+TN)(TN+FN)}}$
Negative Predict Value (NPV)	$\:NPV=\frac{XL}{XL+CL}$
Mean Squared Error (MSE)	$\:MAE=\frac{1}{N}\sum\:_{i=1}^{N}\left|{y}_{e}\left(i\right)-{y}_{a}\left(i\right)\right|$
Normalised Mean Squared Error (NMSE)	$\:NMSE=\frac{\sum\:_{i=1}^{n}{\left({f}_{i}-{y}_{i}\right)}^{2}}{\sum\:_{i=1}^{n}{\left({y}_{i}\right)}^{2}}$
Root Mean Square Error (RMSE)	$\:RMSE=\sqrt{\frac{\sum\:_{i=1}^{N}{\parallel{y}\left(i\right)-\widehat{y}\left(i\right)\parallel}^{2}}{N}}$
Mean Absolute Percentage Error (MAPE)	$\:MAPE=\frac{1}{N}\sum\:_{i=1}^{n}\left|\frac{{y}_{e}\left(i\right)-{y}_{a}\left(i\right)}{{y}_{a}\left(i\right)}\right|\mathrm{*}100$

## Data Availability

Dataset - https://www.kaggle.com/datasets/pengcw1/market-1501/data.
